# Detection of Pesticide Residues Using Three-Dimensional SERS Substrate Based on CNTs/Ag/AgNWs/SiO_2_

**DOI:** 10.3390/s25072316

**Published:** 2025-04-05

**Authors:** Jianjun Ding, Niansong Liu, Ganglin Wang, Naiyu Guo, Chao Sun

**Affiliations:** College of Intelligent Manufacturing, Jianghan University, Wuhan 430056, China; 10107093@jhun.edu.cn (J.D.); liuniansong7105@163.com (N.L.); 16602746073@163.com (G.W.); guo_naiyu@163.com (N.G.)

**Keywords:** surface-enhanced Raman spectroscopy, composite structure, pesticide detection, improving GA-BP

## Abstract

In response to the shortcomings of traditional surface-enhanced Raman spectroscopy (SERS) substrates, such as short shelf life, poor uniformity, and low selectivity, this study innovatively proposed a three-dimensional composite substrate of CNTs/Ag/AgNWs/SiO_2_. This substrate demonstrates excellent SERS enhancement effects, with a detection limit of 10^−12^ mol/L for the probe molecule Rhodamine 6G (R6G) and an enhancement factor (EF) of 8.947 × 10^8^. Further experiments confirmed the substrate’s superior uniformity and stability. The enhancement mechanism was investigated using both experimental methods and the Finite Difference Time Domain (FDTD) approach. When commonly used pesticide thiram was used as the target analyte, the detection limit of the substrate reached 0.1 mg/L, which is significantly lower than the pesticide residue standards of China and the European Union. Additionally, the genetic algorithm (GA)-optimized Back Propagation (BP) neural network was introduced for the quantitative analysis of thiram concentrations. The experimental results indicated that the GA-BP algorithm achieved the training prediction accuracy of 92.5% for thiram, demonstrating good network performance. This method shows good selectivity and has broad application prospects in the detection of toxic chemicals, environmental pollutants, and food additives.

## 1. Introduction

In recent years, with the advancement of global agricultural modernization, the misuse of pesticides has become increasingly prevalent, inflicting incalculable damage on the environment. Consequently, the detection of pesticide residues on crop surfaces is of paramount importance [1]. In the context of daily agricultural production, thiram, a widely used fungicide, has shown significant efficacy in controlling fungal diseases in various crops. However, accidental ingestion by humans can cause severe liver damage. Currently, there are numerous methods for detecting thiram, including high-performance liquid chromatography with ultraviolet detection (HPLC-UV) [2], chemiluminescent enzyme-linked immunosorbent assay (CLIA) [3], and capillary electrophoresis (CE) [4]. While these detection methods are highly accurate, they require complex sample preparation, are time-consuming, and involve cumbersome operations. Therefore, surface-enhanced Raman spectroscopy (SERS) technology offers a non-destructive detection method for pesticides [5], providing an effective approach for rapid pesticide detection.

With the advancement in the field of materials science, the selection of materials for SERS substrates has broadened significantly, and the emergence of nanomaterials has marked a significant leap in the development of SERS technology [6]. Nanomaterials commonly used for the fabrication of SERS substrates are primarily focused on single-metal materials. For metals, certain Raman enhancement effects are produced on their surfaces, and each metal monomer possesses a certain degree of sensitivity. However, not all metals exhibit strong Raman enhancement effects; only gold, silver, copper, and some alkali metals such as lithium and sodium have strong Raman effects, with research on other metals’ Raman effects seeing little progress [7,8]. Lithium and sodium, however, have poor stability and can pose dangers during detection; thus, they are not considered in Raman spectroscopy studies. Yet, with the progress of the times, various new structured materials have developed rapidly, and composite materials are becoming increasingly common. Unlike noble metal materials, materials such as metal oxides, semiconductors, graphene-related materials, and carbon nanotube series materials have weak Raman enhancement effects on molecular substances, but they have stable chemical properties [9,10,11]. When synthesized with metal materials, they can further enhance the Raman signal, making the combination of metal nanomaterials with new materials a new direction for research in SERS substrates.

In current research, noble metal materials continue to be the mainstay in the preparation of SERS substrates. Among them, silver is widely used in the fabrication of SERS substrates because it can produce Surface Plasmon Resonance (SPR) phenomena at laser wavelengths ranging from 300 to 1200 nm [12]. However, silver has poor stability, and the reproducibility of SERS substrates made from pure silver nanostructures is poor, which is not conducive to the reproducibility of the substrate. These issues are not only present in silver nanostructures but are also common problems with pure metal nanostructures [13]. Therefore, to address this problem, researchers have combined silver nanoparticles with non-metals to form new nanostructures to further investigate the enhancement effects of SERS. Substrates formed by the combination of metal nanoparticles and non-metal nanoparticles are more prone to electron transfer, thereby causing an enhancement in the Raman spectroscopy signal. This is because when metal nanoparticles combine with non-metal nanoparticles, an interface is formed between the two, promoting the transfer of electrons. This charge transfer can alter the local electron density, further enhancing the electromagnetic field and thus enhancing the Raman signal. Additionally, the composite structure formed by the combination of metal and non-metal nanoparticles may form specific geometric shapes, creating more “hot spots” where the electromagnetic field intensity is much higher than the surrounding area, thereby greatly enhancing the Raman signal [14]. Under the combined action of these principles, substrates formed by the combination of metal nanoparticles and non-metal nanoparticles exhibit higher sensitivity and selectivity in SERS analysis. For instance, Lv et al. [15] developed a durian-like multilayer core–shell structured SERS substrate (Fe_3_O_4_@Au@Ag@Au) for the detection of pesticide residues in food. This substrate exhibits high sensitivity and good reproducibility, and can effectively detect the pesticide thiram on the surface of apple peels. Zhang [16] and her colleagues synthesized a corn-like Ag@Carbon SERS substrate decorated with metal–organic frameworks (MOFs) by a simple heat treatment of Ag-MOF. The heat treatment transformed Ag into AgNPs, which were better dispersed on the carbonized MOF surface, forming ordered SERS hot spots. Studies have shown that this substrate has detection limits of 2.4 × 10^−8^, 4.8 × 10^−8^, and 2.9 × 10^−8^ for Methylene Blue (MB), Malachite Green (MG), and Crystal Violet (CV), respectively, indicating good application prospects. Park et al. [17] prepared a flexible star-shaped gold nanoparticle SERS substrate by mixing star-shaped gold nanoparticles into a polydimethylsiloxane (PDMS) film. This substrate has a detection limit of 10^−8^ for benzenethiol and an enhancement factor of up to 1.9 × 10^8^, demonstrating excellent performance. Ge et al. [18] developed a three-dimensional (3D) Au/MOF-808(Zr) composite nanostructure using a self-assembly method and found that this structure, as a SERS substrate, has a detection limit as low as 10^−10^ M for the probe molecule R6G and a detection limit of 1.49 × 10^−9^ M for the common fungicide thiram, which can be extended to the sensitive detection of pesticide residues in food and the environment. Zheng et al. [19] combined Au nanoparticles (AuNPs) with zeolite-like imidazolate frameworks (ZIF-8 PCs) to create a novel SERS substrate, which achieved a detection limit of 1.439 × 10⁻^12^ mol/L for the probe molecule 4-mercaptobenzoic acid (4-MBA).

The aforementioned studies have demonstrated the superior performance of multi-dimensional composite SERS substrates, which are increasingly favored by researchers and have shown unique advantages in pesticide detection. However, in most cases, researchers typically focus on the preparation of two-dimensional substrates. Two-dimensional substrates require high precision in laser focusing during substrate preparation. In contrast, three-dimensional substrates can generate more “hot spots” on the surface and increase the substrate’s surface area, leading to a higher enhancement factor. Therefore, designing multi-dimensional composite SERS substrates with better performance will be a future direction for research. Among numerous non-metal materials, carbon possesses strong adsorption capabilities, with multi-walled carbon nanotubes (MWCNTs) exhibiting even greater adsorptive strength, offering more attachment sites and the ability to adsorb a higher number of metal nanoparticles, thereby bringing metal molecules closer together. Furthermore, hydroxylated carbon nanotubes can not only generate certain electromagnetic enhancement effects with metals but also chemical enhancement effects. Consequently, we have introduced multi-walled carbon nanotubes into the preparation of SERS substrates [20].

To ensure compliance with the sensitivity, convenience, and efficiency of pesticide residue detection, this study innovatively combines the advantages of carbon nanotubes and silver to fabricate a CNTs/Ag/AgNWs/SiO_2_ multi-dimensional composite substrate. And R6G is used as the probe molecule to investigate the substrate’s sensitivity, uniformity, stability, and enhancement factor. Subsequently, both experimental and FDTD simulation methods are employed to study the enhancement mechanism of this novel composite substrate, determining the generation method of “hot spots” on the substrate. Furthermore, to further investigate the substrate’s selectivity, a quantitative analysis model for the pesticide thiram is established, utilizing the genetic algorithm to optimize the BP neural network, achieving precise detection of thiram concentrations.

## 2. Materials and Methods

### 2.1. Chemical Materials and Reagents

Multi-walled carbon nanotube dispersion (MWCNT, 14 wt% concentration) was purchased from Nanjing Pioneer Nanomaterials Co., Ltd., Nanjing, China. Silver nitrate (AgNO₃, 0.1 mol/L concentration) was purchased from Beijing Zhongke Erhuan Technology Co., Ltd., Beijing, China. D-(+)-glucose (analytical grade, purity > 99.5%) was purchased from Sigma-Aldrich (Shanghai, China) Trading Co., Ltd., Shanghai, China. Polyvinylpyrrolidone (PVP, 5 wt% concentration, Mw 80000), sodium citrate solution (Na_3_C_6_H_5_O_7_, 98%) and thiram (solid powder) were purchased from Shanghai Aladdin Biochemical Technology Co., Ltd., Shanghai, China. Additionally, anhydrous ethanol and deionized water (DI), used in the experiments, are commercially available products.

### 2.2. Experimental Instruments

The morphology and microstructure of the samples were characterized using a transmission electron microscope (Model JEM-2100plus, JEOL, Tokyo, Japan). The molecular structure of the samples was analyzed by confocal micro-Raman imaging spectroscopy (Model Thermo Dxr2X, Thermo Fisher Technologies, Inc., Waltham, MA, USA), which provides information on vibrational, rotational, and other low-frequency modes through Raman spectra. The surface morphology of the samples was imaged at high resolution using an ultra-high-resolution field emission scanning electron microscope (Model Gemini Sigma 300, Carl Zeiss AG, Jena, Germany). A heat-collecting constant temperature heating magnetic stirrer (Model DF-101S, Shanghai Kohl, Shanghai, China) was employed for stirring solutions under heated conditions, suitable for chemical reactions and sample processing that require temperature control. An ultrasonic cleaner (Model SK5210LHC, Shanghai Kedao, Shanghai, China) was used for cleaning laboratory equipment and dispersing nanomaterials, leveraging the cavitation effect of ultrasound to remove impurities and achieve uniform dispersion. A high-speed freezing centrifuge (Model Sorvall ST16, Thermo Fisher Technologies, Inc., Waltham, MA, USA) was utilized for the separation and purification of components within samples, employing high centrifugal forces to separate solid particles from liquids.

### 2.3. Experimental Procedure

**Figure 1:**
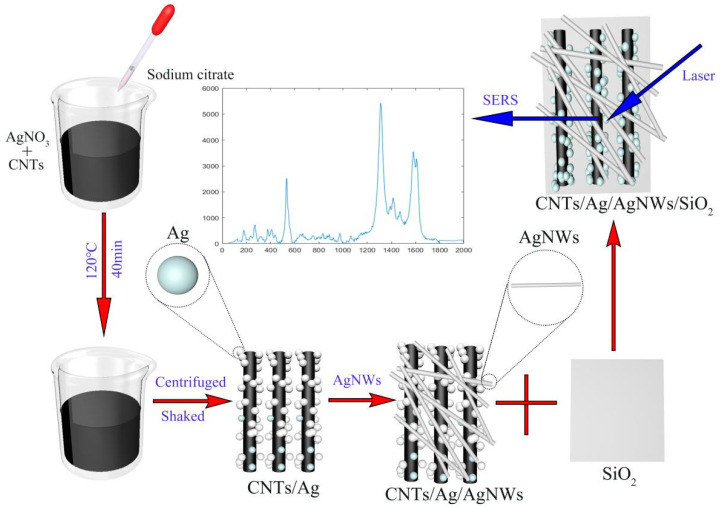
Preparation process of CNTs/Ag/AgNWs/SiO_2_ substrate.

In general, the preparation of the CNTs/Ag/AgNWs/SiO_2_ substrate is divided into three processes: the synthesis of CNTs/AgNPs, the synthesis of AgNWs, and the combination of CNTs/AgNPs with AgNWs, followed by attachment to the glass slide. The preparation process of the CNTs/Ag/AgNWs/SiO_2_ multi-dimensional composite SERS substrate is shown in Figure 1.

### 2.4. Preparation Process of CNTs/AgNPs

In this study, researchers first synthesized Ag nanoparticles of appropriate size according to the method reported in the literature [21], and then prepared CNTs/Ag using the hydrothermal method. Under laboratory-room-temperature conditions (20 to 26 °C), 95 mL of deionized water was taken in a standard sample vial. With a magnetic stirrer rotating at 200 r/min, 5 mL of AgNO_3_ (0.1 M) standard solution, 0.5 mL of multi-walled carbon nanotube dispersion, and 2 mL of sodium citrate Na_3_C_6_H_5_O_7_·2H_2_O (0.0488 M) solution were sequentially added dropwise. The mixed solution was stirred at 200 r/min for 20 min and then heated to 95 °C. The sealed vial was heated for 40 min. After the solution cooled to room temperature, it was centrifuged at a speed of 4500 r/min for 90 min. The resulting turbid sediment was dispersed in 5 mL of deionized water and stored at room temperature for later use.

### 2.5. Preparation Process of AgNWs

Researchers synthesized long silver nanowires (AgNWs) with an average diameter of 45–65 nm and a length greater than 200 µm through the traditional hydrothermal method as proposed by Bari et al. [22]. Under laboratory-temperature conditions (20 to 26 °C), 5 mL of D-(+)-glucose solution (0.1332 M) was mixed with 15 mL of AgNO_3_ solution (0.02 M) and continuously stirred at 120 r/min for 10 min, 5 mL PVP solution with a mass fraction of 16.7% and 15 mL of NaCl solution (0.04 M) were successively added, and the mixture was stirred continuously to ensure uniformity, resulting in a turbid sol. Subsequently, the prepared sol was added to the polytetrafluoroethylene (PTFE)-lined stainless-steel autoclave, and heated in an oven at 160 °C for 22 h. After cooling to room temperature, the solution was transferred to a centrifuge tube and centrifuged at the speed of 2500 r/min for 60 min. The liquid from the upper part of the centrifuge tube was removed, and only the remaining fluffy gray-white precipitate was collected. After adding 5 mL of deionized water, the resulting gray suspension was sonicated (operating frequency of 53 kHz) for 10 min and then stored for later use.

### 2.6. Preparation Process of CNTs/Ag/AgNWs/SiO_2_

**Figure 2:**
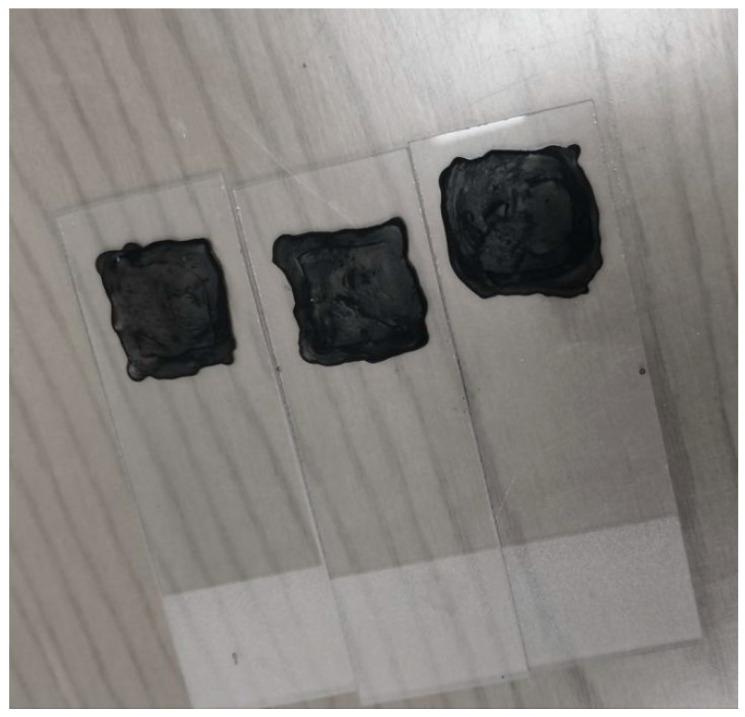
Physical picture of CNTs/Ag/AgNWs/SiO_2_ composite substrate.

Following this, 3 mL of CNTs/AgNPs solution was added to 3 mL of AgNWs suspension, and an ultrasonic cleaner was used to sonicate the mixed solution for minutes (operating frequency of 53 kHz), ensuring uniform mixing. The CNTs/Ag/AgNWs suspension was then drop-cast onto cleaned glass slides, allowed to air dry naturally, and subsequently stored in a sealed environment at 4 °C for future use, yielding the CNTs/Ag/AgNWs/SiO_2_ substrate. Figure 2 is the physical image of the CNTs/Ag/AgNWs/SiO_2_ substrate prepared by the experiment.

### 2.7. Sensitivity Testing

The experiment utilized R6G as a probe molecule to evaluate the Raman performance of the CNTs/Ag/AgNWs/SiO_2_ composite substrate. A total of 0.2 mL of R6G solution with a concentration of 10^−8^ mol/L was drop-cast onto the prepared CNTs/Ag/AgNWs/SiO_2_ substrate, and the SERS analysis was conducted after the liquid dried. For comparison, under the same fixed R6G concentration, the SERS performance of Ag and Ag/AgNWs was also assessed using a similar method. Considering the simplicity of the article, the specific preparation process of Ag sol and Ag/AgNWs is shown in the Supplementary Materials attached file.

### 2.8. SERS Detection of Thiram

To investigate the actual detection performance of the substrate for pesticide residues, we selected the commonly used pesticide thiram as the target analyte and prepared thiram ethanol solutions with concentrations of 10 mg/L, 5 mg/L, 1 mg/L, 0.1 mg/L, and 0.01 mg/L using anhydrous ethanol as the solvent. Different concentrations of thiram solutions were drop-cast onto the prepared CNTs/Ag/AgNWs/SiO_2_ substrate, and the SERS analysis was conducted after the liquid dried.

### 2.9. Characterization

Transmission electron microscopy (TEM) and high-resolution transmission electron microscopy (HRTEM) images were obtained using a JEM-2100plus operating at an acceleration voltage of 200 kV. Raman spectra were recorded using a confocal micro-Raman imaging spectroscopy (Thermo DXR2x) equipped with a 633 nm laser, with a laser power of 392 mW and a data acquisition time of 20 s.

## 3. Results and Discussion

### 3.1. Characterization of CNTs/Ag/AgNWs/SiO_2_ Composite Substrate

**Figure 3:**
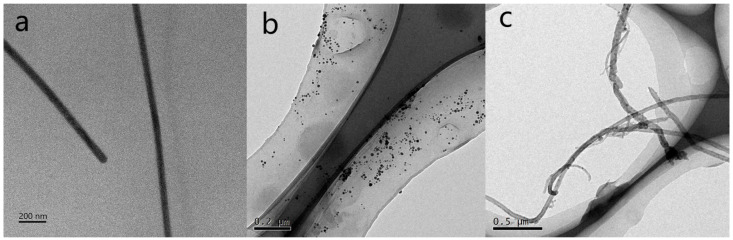
(**a**) TEM characterization of AgNWs nanoparticles, (**b**) TEM characterization of CNTs/Ag nanoparticles, (**c**) TEM characterization of CNTs/Ag/AgNWs/SiO_2_.

During the preparation of the CNTs/Ag/AgNWs/SiO_2_ composite substrate, researchers employed the hydrothermal synthesis method to utilize the reducing property of glucose to reduce Ag^+^ to AgNPs. Subsequently, PVP was used as a capping agent, ultimately forming AgNWs. The TEM characterization results obtained using a transmission electron microscope are shown in Figure 3a. Figure 3a shows that the linear structure of AgNWs was successfully fabricated. Measurements taken with Nano Measurement software (version 1.2.5) revealed that the average diameter of the AgNWs is 45–65 nm, with lengths greater than 200 µm, and an average interspacing of 1.4 nm. Thereafter, Ag nanoparticles were attached to CNTs via the hydrothermal method to form the CNTs/Ag structure, as evidenced by the TEM characterization shown in Figure 3b. Subsequently, the researchers mixed the AgNWs nanoparticle solution with the CNTs/Ag and then drop-cast the mixture onto a SiO_2_ substrate. As shown in Figure 3b, AgNWs are well integrated with CNTs/Ag, confirming that the CNTs/Ag/AgNWs were successfully prepared.

**Figure 4:**
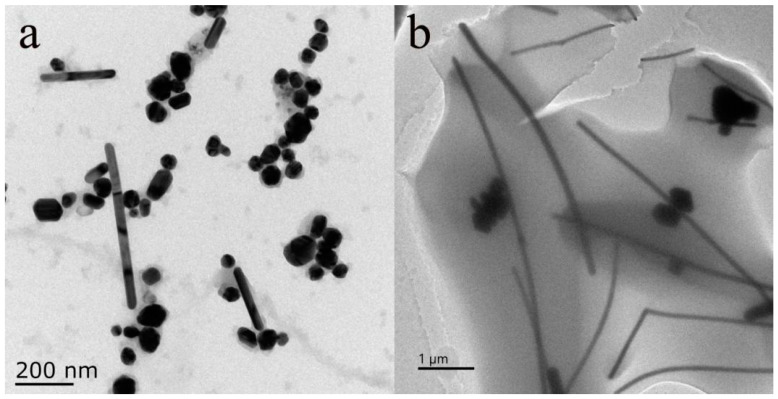
(**a**) TEM characterization of AgNWs without PVP. (**b**) TEM characterization of AgNWs with PVP.

To further investigate whether PVP has an impact on the preparation of AgNWs, under laboratory conditions, we set the reaction conditions at 160 °C for 13 h for the experiment. Figure 4a displays the TEM characterization of AgNWs formed without the addition of PVP during the synthesis process. Measurements of the AgNWs dimensions using Nano Measurement software revealed that the diameters of AgNWs ranged from 35 to 192 nm, and there were few AgNWs exceeding 200 µm in length. In addition, a significant number of silver nanoparticles were observed as by-products, with diameters of approximately around 40 nm. Subsequently, PVP was introduced into the reaction solution while keeping all other solution concentrations and amounts unchanged. The TEM characterization of the product after the reaction is shown in Figure 4b. It can be seen that the majority of the reaction products were uniformly sized and shaped AgNWs, which showed a significant change in dimensions compared to the products without PVP. Although a small number of silver nanoparticles were still produced after the addition of PVP, this was likely due to PVP not capping all the formed Ag or the capping occurring too rapidly, resulting in a small number of silver nanoparticles or even short silver nanorods remaining after the reaction was terminated. This study indicates that PVP has a good capping effect on the synthesis of AgNWs, promoting their formation and reducing the appearance of by-products.

### 3.2. SERS Performance of CNTs/Ag/AgNWs/SiO_2_ Composite Substrate

To further investigate the SERS performance of the synthesized CNTs/Ag/AgNWs/SiO_2_ composite and to enhance the credibility of the experimental results, the researchers introduced the common probe molecule R6G to evaluate the sensitivity of the substrate.

**Figure 5:**
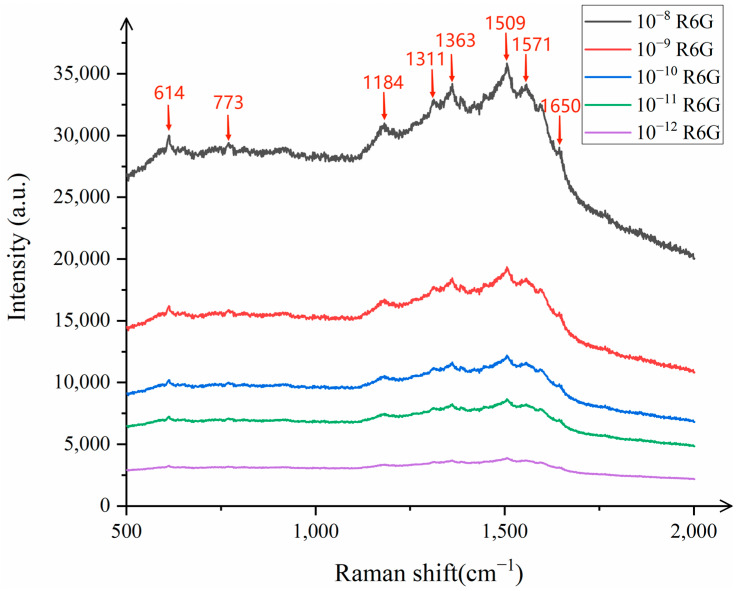
Raman spectra of R6G with different concentrations.

**Figure 6:**
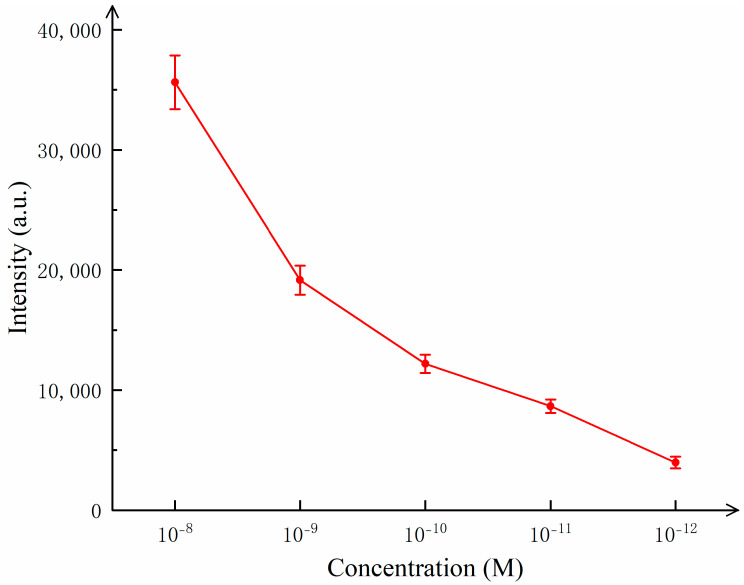
Raman signal intensity changes with concentration.

As shown in Figure 5, the distinct characteristic peaks of R6G can be clearly observed at 614 cm^−1^, 773 cm^−1^, 1184 cm^−1^, 1311 cm^−1^, 1363 cm^−1^, 1509 cm^−1^, 1571 cm^−1^, and 1650 cm^−1^, indicating that the SERS successfully detected the characteristic information of R6G. To further explore the lowest detection limit of R6G, the substrate was tested with R6G concentrations of 10^−12^ mol/L and 10^−13^ mol/L. The results showed that the characteristic peaks of R6G at 10^−13^ mol/L were almost non-existent, while the characteristic peaks of R6G at 10^−12^ mol/L were clearly visible, demonstrating that the detection limit of the composite substrate for R6G reached 10^−12^ mol/L, which has excellent sensitivity. Figure 6 illustrates the variation in the detected Raman signal intensity with the concentration of R6G.

### 3.3. Calculation of Enhancement Factor of CNTs/Ag/AgNWs/SiO_2_ Composite Substrates

To evaluate the enhancement capability of the substrate, this study measured the enhancement factor of the target molecules on the substrate, thereby allowing for a quantitative and intuitive understanding of the substrate’s enhancement effect. The experiment employed a method for calculating the enhancement factor as reported in reference [23], with the calculation method shown in Equation (1).

(1) $$SERS\_EF=\frac{I_{SERS}/C_{SERS}}{I_{R\mathrm{aman}}/C_{Raman}}$$

In the equation, $I_{SERS}$ represents the Raman intensity detected after the probe molecules are adsorbed onto the enhancing substrate, $I_{R\mathrm{aman}}$ represents the Raman intensity when only the probe molecules are detected without the substrate, $C_{SERS}$ represents the concentration of probe molecules adsorbed on the substrate, and $C_{Raman}$ represents the concentration of probe molecules not adsorbed on the substrate.

**Figure 7:**
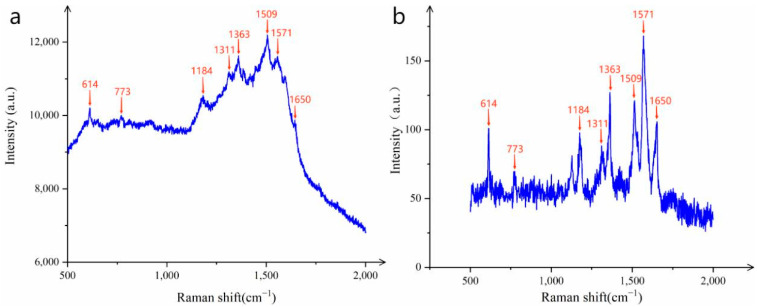
(**a**) R6G (10^−10^ mol/L) adsorbed on the substrate. (**b**) The Raman signal of R6G (10^−3^ mol/L) without the substrate test.

As shown in Figure 7, taking the characteristic peak at 1509 cm^−1^ of R6G’s Raman shift as an example, the Raman intensity of R6G with a concentration of 10^−10^ mol/L on the CNTs/Ag/AgNWs/SiO_2_ substrate was measured to be 12,035.642, while the Raman signal intensity of a R6G solution with a concentration of 10^−3^ mol/L on the SiO_2_ glass slide was 135.492. Therefore, in the calculation of the enhancement factor, $I_{SERS}$ = 12035.642, $I_{Raman}$ = 135.492, $C_{SERS}$ = 10^−10^ mol/L, and $C_{R\mathrm{aman}}$ = 10^−3^ mol/L. Consequently, $SERS\_EF=\frac{I_{SERS}/C_{SERS}}{I_{Raman}/C_{Raman}}$ = $\frac{12035.64211/10^{-10}}{135.492/10^{-3}}$ = $8.882\times 10^{8}$. Subsequently, we calculated the enhancement factors for nine characteristic peaks and then for all Raman shift data. The results are shown in Table 1. From Table 1, it can be seen that the enhancement factors for the two sets of data are quite similar, indicating that the substrate has excellent performance.

### 3.4. Exploration of the Sensing Mechanism of CNTs/Ag/AgNWs/SiO_2_ Composite Substrate

**Figure 8:**
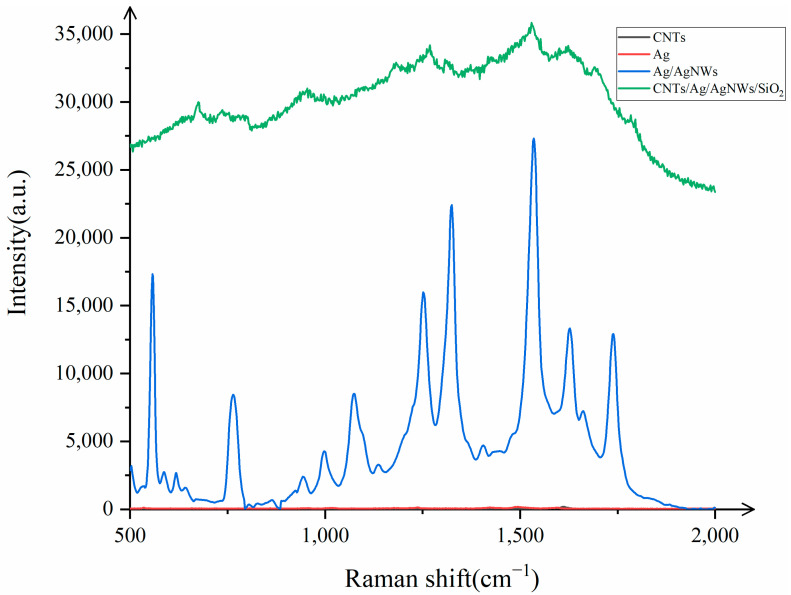
Comparison of four different substrate Raman signals.

Three-dimensional substrates used for SERS detection are typically composed of various materials, each of which contributes differently to the enhancement in the target analyte. Therefore, to better investigate the enhancement mechanisms of the substrate, this study compares the enhancement effects of multi-walled carbon nanotubes, silver colloid, Ag/AgNWs, and CNTs/Ag/AgNWs/SiO_2_. A probe molecule concentration of 10^−8^ mol/L of R6G was used to analyze the enhancement mechanism of CNTs/Ag/AgNWs/SiO_2_. The test results are shown in Figure 8.

It is evident from Figure 8 that when AgNWs are used as the substrate for Raman detection of R6G (10^−8^ mol/L), only the characteristic peak of the multi-walled carbon nanotubes at 1591 cm^−1^ is detected, with a small peak signal intensity, indicating poor performance. Subsequently, after attaching Ag nanoparticles to the surface of AgNWs and performing Raman detection on R6G, the signal intensity increases to some extent. This is because the attachment of Ag nanoparticles generates electromagnetic enhancement with AgNWs, and electromagnetic enhancement also occurs between the silver nanoparticles themselves. The combined effect results in Ag/AgNWs exhibiting good Raman enhancement, with the Raman intensity being 5.15 times (on average) that of AgNWs when detecting R6G. Finally, when we combine the core–shell structure of Ag/AgNWs with carbon nanotubes, the CNTs/Ag/AgNWs/SiO_2_ composite substrate shows a Raman intensity that is 1.48 times (on average) that of Ag/AgNWs when detecting R6G. This is attributed to the charge transfer between the multi-walled carbon nanotubes and Ag/AgNWs, generating additional chemical enhancement within the substrate, which, in turn, leads to a certain degree of enhancement in the Raman signal during detection.

The Raman enhancement by the CNTs/Ag/AgNWs/SiO_2_ composite substrate can be attributed to three distinct mechanisms: (1) enhancement caused by carbon nanotubes, which includes both the intrinsic enhancement in the probe molecules by the carbon nanotubes themselves and the enhancement due to charge transfer between the carbon nanotubes and the probe molecules; (2) enhancement caused by Ag/AgNWs, which involves the enhancement in the probe molecules by Ag nanoparticles, silver nanowires, and the interaction between Ag nanoparticles and AgNWs, as well as the enhancement caused by charge transfer between Ag/AgNWs and the probe molecules; (3) enhancement caused by charge transfer between multi-walled carbon nanotubes and Ag/AgNWs.

The first two reasons involve both electromagnetic enhancement (EM) and chemical enhancement (CM), while the third is attributed solely to CM. To investigate the contribution of each component to the Raman enhancement factor, this study calculated the enhancement factors for CNTs, Ag/AgNWs, and CNTs/Ag/AgNWs/SiO_2_ substrates separately to explore the contribution rate of each part to the Raman enhancement. After calculations, it was found that the enhancement factors for CNTs, Ag/AgNWs, and CNTs/Ag/AgNWs/SiO_2_ substrates were 2.33, and the enhancement factors for CNTs and Ag/AgNWs were calculated as a percentage of the enhancement factor for CNTs/Ag/AgNWs/SiO_2_ to determine the contribution rate of the four substrates. It was observed that the contribution rate of CNTs was nearly 0, the contribution rate of Ag/AgNWs was 83.86%, and the remaining enhancement effect, attributed to other factors, accounted for 16.14%. This phenomenon is due to the fact that after the attachment of Ag nanoparticles to the surface of AgNWs, the electromagnetic effect of the substrate is enhanced and becomes the dominant component, while the chemical enhancement effect produced by the charge transfer of carbon nanotubes is far smaller than the electromagnetic enhancement generated by Ag/AgNWs.

### 3.5. Exploration of the Uniformity of CNTs/Ag/AgNWs/SiO_2_ Composite Substrate

**Figure 9:**
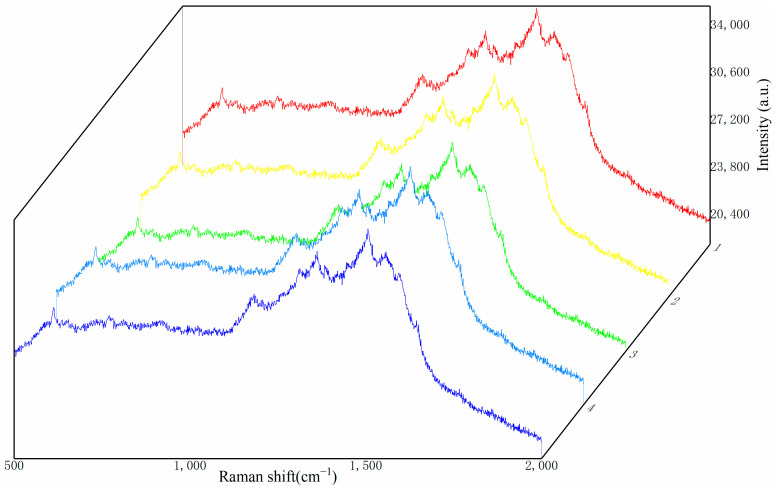
SERS signals of CNTs/Ag/AgNWs/SiO2 substrate at five different positions. (In order to distinguish, different colors represent different positions).

In practical applications, it is often not convincing to only test a small area of the substrate. Due to the randomness of the testing, the selected area is not always the same, which places a high demand on the uniformity of the substrate. To investigate the uniformity of the substrate, this experiment involved adsorbing R6G at a concentration of 10^−8^ mol/L on the prepared composite substrate and randomly selecting five positions for SERS measurements. The results are shown in Figure 9.

Figure 9 demonstrates that Raman detection at five different positions on the substrate yields clear characteristic peaks, with the Raman shifts corresponding to these peaks being essentially identical, with deviations within the range of 1–2 cm^−1^, which is within the acceptable margin of error. The relative consistency in the Raman signal intensity across the five positions preliminarily indicates that the prepared substrate has good uniformity. To obtain a clearer assessment of the substrate’s uniformity, this study introduces the relative standard deviation (RSD) as an evaluation criterion and employs Equation (2) to calculate the RSD for several major characteristic peaks of R6G.

(2) $$RSD=\sqrt{\frac{\sum_{i=1}^{n}{\left(x_{i}-\bar{x}\right)}^{2}}{n-1}}\times \frac{1}{\bar{x}}\times 100\%$$

In the equation, $x_{i}$ represents the Raman signal intensity of the characteristic peak at 1509 cm^−1^ at the i-th position, and $\bar{x}$ represents the average Raman signal intensity of the 1509 cm^−1^ peak at five different positions, *n* = 5. Six major characteristic peaks of R6G were selected, and their RSD values are presented in Table 2. The results show that although there are some differences in the Raman signal intensities at different positions, the RSD of the signal intensities corresponding to the same Raman shift for the characteristic peaks does not exceed 3.5%, proving that the substrate has good uniformity.

### 3.6. Exploration of the Stability of the CNTs/Ag/AgNWs/SiO_2_ Composite Substrate

**Figure 10:**
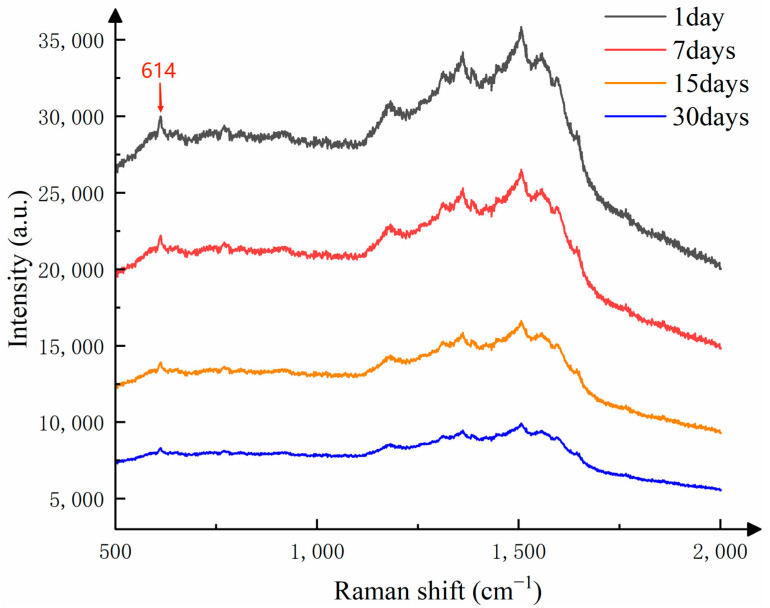
Raman signal comparison of the substrate after storage for a certain period of time.

To investigate the stability of the substrate when stored at room temperature (20–25 °C), the researchers spotted the substrate surface with the same volume of 10⁻^8^ mol/L R6G solution and then detected its Raman signal after storage for 1, 7, 15, and 30 days. The detection results are shown in Figure 10.

**Figure 11:**
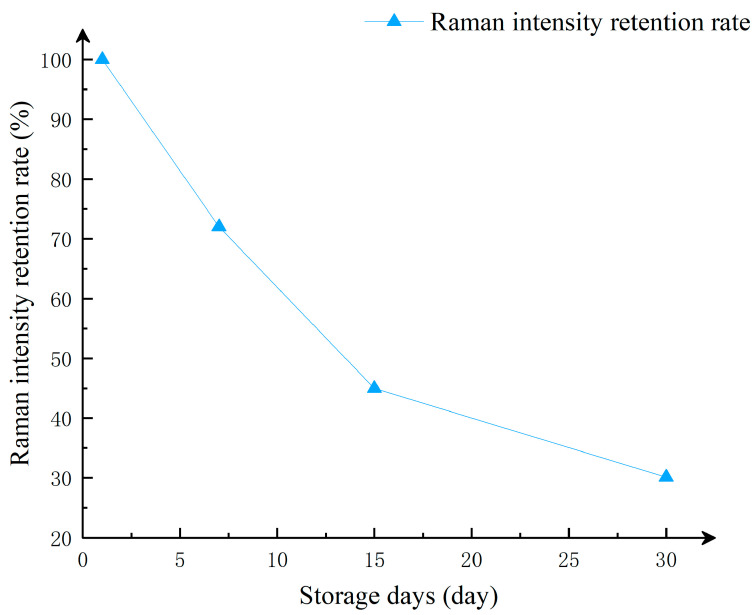
The relationship between the preservation percentage of Raman signal at 614 cm^−1^ and the preservation time of the substrate.

In this study of substrate stability, we used the Raman signal intensity detected on the first day as the standard, and studied the change in Raman intensity of the substrate stored at different times on the Raman characteristic peak corresponding to the Raman shift of 614 cm^−1^. As shown in Figure 9, after one week of storage, the intensity of the Raman signal decreased. This is because the silver nanoparticles and silver nanowires in the substrate, when exposed to air for a period of time, undergo oxidation and form an oxide layer, which affects the optical properties of the nanoparticles and leads to a reduction in Raman signal intensity. Additionally, long-term storage can cause aggregation of silver nanoparticles, and the aggregated state also affects the local field enhancement effect, thereby weakening the Raman signal intensity. Even after one month of storage, the detected Raman signals still show distinct characteristic peaks. As shown in Figure 11, although the Raman signal intensity of the substrate decreases slowly over time, it still maintains a strong Raman signal. Therefore, the CNTs/Ag/AgNWs/SiO_2_ composite substrate exhibits good stability and is capable of meeting the requirements for practical applications.

### 3.7. SERS Quantitative Detection of Thiram

**Figure 12:**
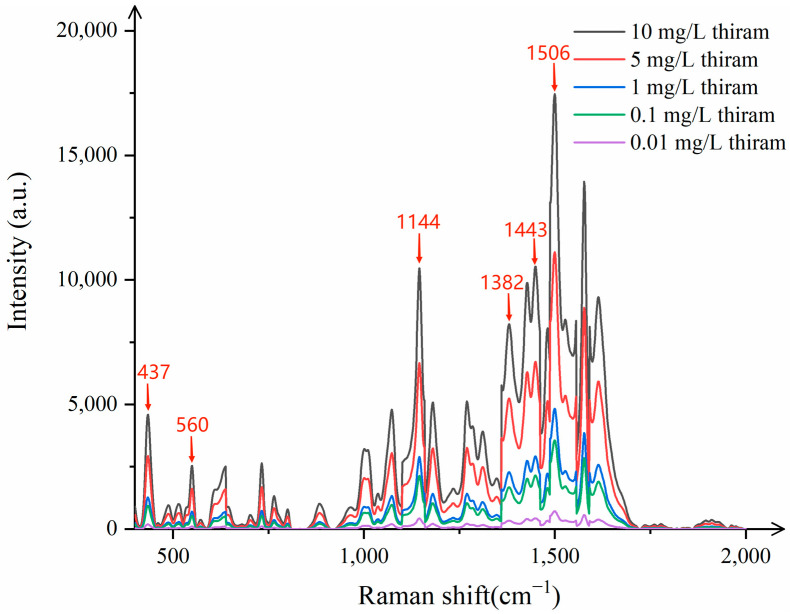
Raman spectra of thiram with different concentrations.

In order to evaluate the practical applicability of CNTs/Ag/AgNWs/SiO_2_ as SERS substrate, we used the common pesticide thiram as the test object, and used Raman spectrometer to detect the Raman spectra of different concentrations of thiram, as shown in Figure 12.

Figure 12 indicates that when Raman detection is performed on thiram ethanol solutions with concentrations ranging from 0.01 mg/L to 10 mg/L, clear characteristic peaks are present at 437 cm^−1^, 560 cm^−1^, 1144 cm^−1^, 1382 cm^−1^, 1443 cm^−1^, and 1506 cm^−1^, even at concentrations as low as 0.1 mg/L, which almost coincide with the standard characteristic peaks of thiram. Upon testing thiram at the concentration of 0.01 mg/L, the peaks become indistinguishable. Therefore, the minimum detectable concentration of thiram by this substrate is 0.1 mg/L, meeting the actual detection needs.

### 3.8. Mixed Detection of Thiram and Diquat Pesticide

In the actual detection of pesticides, multiple pesticides often coexist, and the interference from other matrices needs to be considered. Therefore, it is necessary to further verify the detection capability of the substrate for mixtures of multiple pesticides. To this end, this study designed two experiments to detect the Raman spectra of mixed pesticide solutions to evaluate the applicability of the CNTs/Ag/AgNWs/SiO_2_ composite substrate in complex samples.

**Figure 13:**
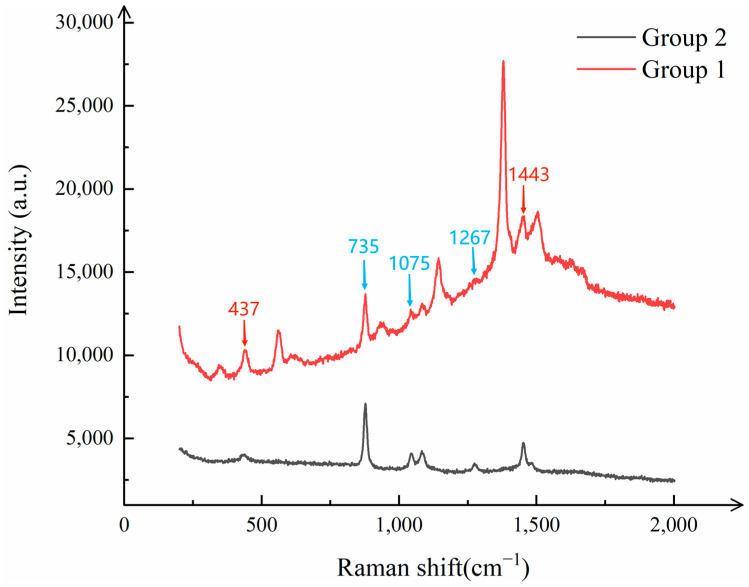
Raman spectra of mixed pesticide detection (Red represents the characteristic peak of thiram, blue represents the characteristic peak of diquat).

Specifically, two mixed pesticide solutions were prepared: one containing 6 mg/L of thiram ethanol solution and 4 mg/L of paraquat ethanol solution, which was defined as “Group 1”; and the other containing 4 mg/L of thiram ethanol solution and 6 mg/L of paraquat ethanol solution, which was defined as “Group 2”. To ensure the uniformity of the mixtures, both solutions were subjected to ultrasonic oscillation before being drop-cast onto the CNTs/Ag/AgNWs/SiO_2_ composite substrate. After the liquid on the substrate dried, Raman detection was performed. The detection results of the mixed pesticides are shown in Figure 13.

As can be clearly observed from Figure 13, the Raman spectra of the two mixed pesticide solutions exhibit distinct characteristic peaks. These peaks are well matched to the characteristic Raman shifts in thiram and paraquat, such as at 437 cm^−1^, 735 cm^−1^, 1075 cm^−1^, 1267 cm^−1^, and 1443 cm^−1^. This indicates that the CNTs/Ag/AgNWs/SiO_2_ composite substrate is capable of effectively identifying and differentiating the characteristic information of both pesticides in the presence of their mixture, thereby enabling precise detection of mixed pesticides.

## 4. FDTD Simulation and Result Analysis

### 4.1. FDTD Simulation

**Figure 14:**
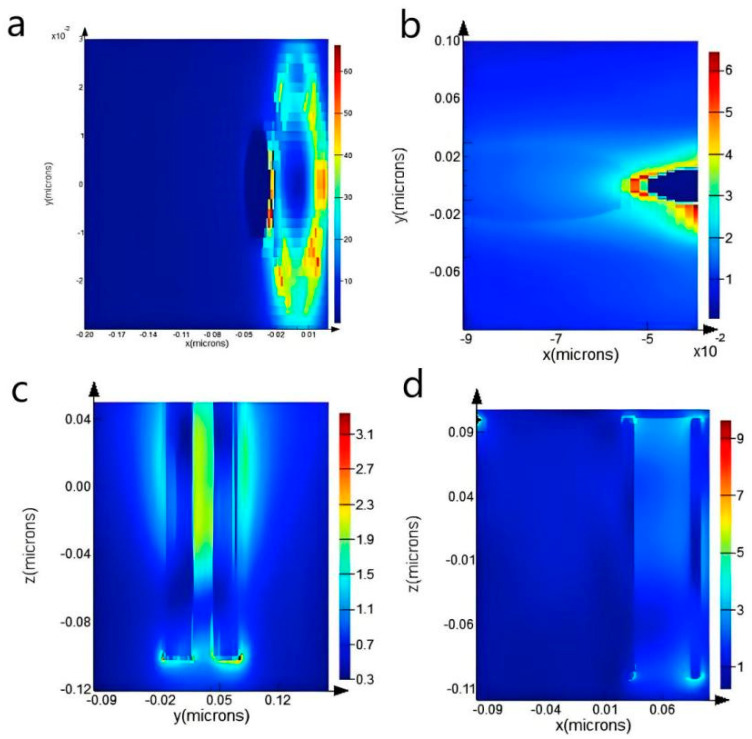
(**a**) Simulation results of R monitor. (**b**) Simulation results between AgNPs and AgNWs. (**c**) Simulation results of AgNWs-2 monitor. (**d**) Simulation results of Ag-C monitor.

In order to further explore the enhancement effect of the CNTs/Ag/AgNWs/SiO_2_ composite substrate, the finite difference time domain method was used to simulate the electric field distribution of different nanostructures. The simulation results are shown in Figure 14.

From the simulation results, it can be observed that there are four types of “hot spots” on the CNTs/Ag/AgNWs/SiO_2_ composite substrate: “hot spots” generated between silver nanoparticles and carbon nanotubes, “hot spots” generated between silver nanoparticles and silver nanowires, “hot spots” generated between silver nanowires, and “hot spots” generated between silver nanowires and carbon nanotubes. It can be deduced that, in theory, the CNTs/Ag/AgNWs/SiO_2_ composite substrate is capable of producing strong electromagnetic fields and exhibits good SERS effects.

### 4.2. Prediction of Pesticide Concentration Using the Improving GA-BP Neural Network Model

After detecting the thiram pesticides with the CNTs/Ag/AgNWs/SiO_2_ substrate, we obtained the Raman signals and their corresponding concentrations. To further improve the accuracy of predicting pesticide concentrations, we combined the pesticide detection process with machine learning, employing the improving GA-BP neural network model to achieve quantitative detection of the concentrations of thiram pesticide.

Genetic algorithms integrate Darwin’s theory of evolution and Mendel’s genetics, searching for the optimal individuals through selection, crossover, mutation, and other genetic evolutionary methods. They offer a broader search range, stronger robustness, and are more efficient and practical in selecting the best solutions, with very wide applications across various mainstream industries [24].

**Figure 15:**
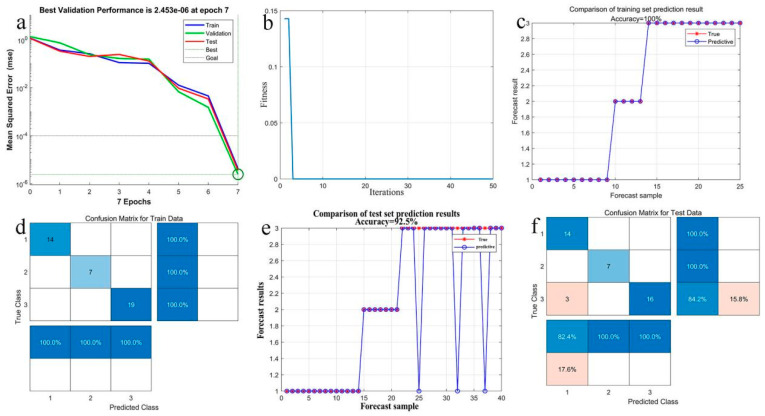
Test results of each index of thiram. (**a**) the best training performance, (**b**) the best fitness value, (**c**) the prediction accuracy of training set, (**d**) the prediction confusion matrix of training set, (**e**) the prediction accuracy of test set, (**f**) the prediction confusion matrix of test set.

For thiram, we trained and tested the GA-BP neural network model using all the Raman signals detected at concentrations of 0.1 mg/L, 1 mg/L, and 5 mg/L as inputs, with the concentration as the output. The test results of each index of thiram are shown in Figure 15.

In order to verify that the improving GA-BP neural network is superior to other machine learning methods, this study compares it with mathematical fitting results and other published intelligent algorithms such as mathematical fitting (MF), BP neural network, traditional GA-BP, and LSSVM. The comparison results are shown in Table 3.

Table 3 clearly demonstrates that the improving GA-BP neural network proposed in this study performs more admirably in the prediction experiments of pesticide concentrations. The primary reason for this outcome is attributed to the global search capability of the genetic algorithm. For the complex dataset detected, there is a lack of obvious correlations between the data, and the data are widely distributed, which undoubtedly increases the challenge and complexity in the global optimization process for the algorithm. The genetic algorithm is capable of efficiently utilizing existing excellent solutions while exploring new ones, thus avoiding becoming trapped in local optima and accelerating the convergence process. It is these characteristics that enable the GA-BP neural network to exhibit superior network performance in predicting pesticide concentrations.

## 5. Conclusions

This study utilized silver and multi-walled carbon nanotubes as materials, employing the hydrothermal method to attach silver nanoparticles to carbon nanotubes, which were then combined with prepared AgNWs and attached to SiO_2_ wafers to fabricate the CNTs/Ag/AgNWs/SiO_2_ SERS composite substrate. The substrate achieved the minimum detection limit of 10^−12^ mol/L for R6G, with the enhancement factor of 8.947 × 10^8^. Compared to the substrate without AgNWs, the detection limit for R6G was reduced by an order of magnitude, and the enhancement factor was increased by 7.28 times, demonstrating good uniformity and stability. Subsequently, the enhancement mechanism of the substrate was studied from both experimental and FDTD simulation perspectives. Raman detection experiments were conducted on the common pesticide thiram, and the results showed that the composite substrate had a detection limit of 0.1 mg/L for thiram, meeting practical requirements. The BP neural network optimized by the genetic algorithm was used to predict the concentration of the pesticide thiram, and the results indicated that the improving GA-BP algorithm outperformed the traditional BP neural network in predicting pesticide concentrations, satisfying practical detection demands.

## Figures and Tables

**Table 1:** EF calculation of flower-like silver-flexible sponge.

**Selected Characteristic Peaks**	**Signal Number**	**Average** EF	**The Standard Deviation of ** EF
9 characteristic peak displacements	9	8.947 × 10^8^	2.16
All Raman shifts	900	8.673 × 10^8^	3.27

**Table 2:** The relative standard deviations (RSDs) of the main characteristic peaks of R6G (10^−8^ mol/L) absorbed on CNTs/Ag/AgNWs/SiO_2_ composite substrate.

Raman shift (cm^−1^)	614	773	1184	1311	1363	1509
RSD (%)	1.29	0.61	3.15	2.13	1.37	2.29

**Table 3:** Accuracy comparison between improving GA-BP and other algorithms.

**Algorithm Type**	**MF**	**BP**	**Traditional GA-BP**	**LSSVM**	**Improving GA-BP**
The prediction accuracy of thiram	89.34%	85%	87.1%	90.83%	92.5%

## Data Availability

Data will be made available on request.

## Supplementary Materials

The following supporting information can be downloaded at: https://www.mdpi.com/article/10.3390/s25072316/s1.
