# Supplementary material for: Detection of Pesticide Residues Using Three-Dimensional SERS Substrate Based on CNTs/Ag/AgNWs/SiO2
Source: Sensors (Basel). 2025 Apr 5;25(7):2316. doi: 10.3390/s25072316 (PMC11991087; doi:10.3390/s25072316)
Supplement: Supplementary file 1 [file sensors-25-02316-s001.zip › sensors-3528512-supplementary.pdf]

Supplementary material

Preparation of Ag sol

To prepare silver nanosol, 100 mL of $AgNO_3$ solution(0.005 M) was added to a 250 mL beaker, and an appropriately sized magnetic stirrer was placed inside the beaker. The beaker was then sealed with plastic wrap and fixed in the center position of a heat-collecting magnetic stirrer hotplate. The target oil temperature of the stirrer was set to 120 °C, and the stirring speed was set to 120 r/min. When the temperature rises to 95 °C, sodium citrate solution (2mL, mass fraction of 1 %) is quickly added to the beaker, and the beaker is heated for 30 min while stirring. After the reaction was complete, the beaker was removed from the heat-collecting magnetic stirrer hotplate and allowed to cool naturally to room temperature. Then, 10 mL of the liquid was transferred to a centrifuge tube, and the centrifuge was set to rotate at 1600 r/min for 15 minutes. After centrifugation, the supernatant was removed using a pipette, leaving only the colloidal lower layer. A pipette was used to take 1 mL of the bottom sol from the centrifuge tube, which was then diluted with 5 mL of deionized water and sonicated for 10 minutes using an ultrasonic cleaner before being sealed and stored in a test tube for later use.

Preparation of Ag/AgNWs

Using a rubber bulb pipette, 3 mL of AgNWs was extracted and added to a centrifuge tube, followed by the addition of 3 mL of silver sol. An ultrasonic cleaner was then used to sonically agitate the mixed solution, ensuring uniform mixing of the two solutions. Subsequently, the prepared Ag/AgNWs mixture was sealed and stored in a refrigerator at 4 °C for future use.
